# First experience with VITOM eagle in micro-laryngeal surgery on 3D-printed laryngeal models: important improvements in exoscopic technology

**DOI:** 10.1007/s00405-025-09293-0

**Published:** 2025-03-14

**Authors:** E. Errico, S. Mauramati, G. Bertino, E. Robiolio, M. V. Veneroni, M. Benazzo, R. Gelli

**Affiliations:** Department of Otorhinolaryngology, University of Pavia, Foundation IRCCS Policlinico San Matteo, Pavia (PV), Italy

**Keywords:** Exoscopy, 3D, Otolaryngology, VITOM^®^, Micro-laryngeal surgery, TOLMS

## Abstract

**Purpose:**

The Karl Storz Company recently introduced a new exoscopic system equipped with new features and improvements compared to older counterparts. The endpoints of this study are to evaluate the new VITOM^®^ Eagle system and compare it to the older VITOM^®^ 3D for micro-laryngeal surgery, to analyze the new features of the latest Karl Storz’s product and determine whether these upgrades provide a significant advantage to surgery of the laryngeal district.

**Methods:**

Participants simulated CO_2_ Transoral Laser Microsurgery (CO_2_ TOLMS) procedures on 3D-printed laryngeal models firstly with both the VITOM^®^ 3D and VITOM^®^ Eagle. At the end of both sessions 2 questionnaires were administered: the first generic 3-item questionnaire comparing evaluating exoscopic and microscopic technology, the second questionnaire involved 8 items related to device quality.

**Results:**

Overall the new VITOM^®^ Eagle resulted superior to the older VITOM^®^ 3D, there were statistically significant differences in scores evaluating the image quality (*p* = 0.002), magnification rate (*p* = 0.002), and luminance (*p* = 0.047). In addition, according to questionnaires results, senior Otolaryngologists appeared to be slightly less inclined to a complete transition to exoscopy when compared to young surgeons.

**Conclusions:**

The results of this study demonstrate how the new VITOM^®^ Eagle significantly outperforms the older VITOM^®^ 3D. The upgrades discussed, have the potential of leading to better surgical outcomes by providing surgeons with better tools to navigate complex anatomical districts. Furthermore, results from this study highlighted how exoscopy is affecting the surgical training of young surgeons.

**Supplementary Information:**

The online version contains supplementary material available at 10.1007/s00405-025-09293-0.

## Introduction

Exoscopic technology has emerged in the past decade as a novel tool for surgical visualization and magnification, designed to replace traditional operating microscopes in various head and neck surgeries, ultimately reducing associated costs. The Karl Storz company (Tuttlingen, Germany) recently introduced the VITOM^®^ Eagle exoscope, which combines the 4 K resolution of the previous VITOM^®^ 3D model with new 3D capabilities and several additional benefits, these new features account for a big technological step-up in exoscopic technology that will not only enhance its application in procedures in which its use has already been tested, but also they will advance the possibility for broadening its applications in the Otolaryngological district. The benefits and potential of exoscopic technology in laryngeal surgeries have already been described in literature [1–7], further advancements in this technology strengthen the hypothesis that exoscopic surgery could potentially overcome conventional operating microscopes in minimally invasive laryngeal surgery in the near future. This experimental study was carried out in a simulation setting similar to the one used in previous studies [2, 8], with the aim to evaluate the new benefits of exoscopic surgery with VITOM^®^ Eagle exoscope and compare them to the older model (VITOM^®^ 3D) in laryngeal surgeries, using 3D-printed laryngeal models. To our knowledge, this is the first study that attempts to evaluate the new advantages offered by VITOM^®^ Eagle.

## Materials and methods

A total of 10 participants, 5 Senior Otolaryngologists (ENT) and 5 residents in Otolaryngology tested both the new VITOM^®^ Eagle + ARTip™ system and the older VITOM^®^ 3D + Versacrane™ both equipped with a micromanipulator and CO_2_ laser on 3D-printed laryngeal anatomical models in the Surgical Simulation Laboratory of the Integrated Unit of Experimental Surgery, Advanced Microsurgery and Regenerative Medicine of the University of Pavia, Italy. Attendees were asked to simulate CO_2_ Transoral Laser Microsurgery (CO_2_ TOLMS) [9] procedures. After performing the procedures with each system, participants were asked to fulfill two questionnaires (questionnaires available as online resource supplementary material). The first questionnaire involved 3 general questions on microscopic/exoscopic surgery in the laryngeal district: 1 question regarding the frequency of the need to convert the procedure to an operating microscope (OM) on a 4-point Likert scale (1 - Always, 2– Frequently, 3– Almost never, 4– Never) and 2 questions comparing VITOM^®^ Eagle to OM and to VITOM^®^ 3D respectively, on a 4-point Likert scale (1 - Inferior, 2 - Equal, 3– Superior, 4– Very superior). Afterwards candidates were administered the second questionnaire with 8 device-specific questions comparing the VITOM^®^ 3D and the VITOM^®^ Eagle on a 4-point Likert scale (1 - not acceptable, 2 - acceptable, 3– good, 4– very good), similarly to De Virgilio et al. [2].

No evaluation for approval from the Ethical Committee was necessary, as this study did not involve experimentation on neither humans nor animals. Patients that might have been involved in the images displayed in this study, signed an informed consent form for disclosure of appropriate personal data for scientific purposes.

### Statistical analysis

Answers of the first questionnaire were calculated in terms of percentages of choice and displayed in a cumulative descriptive analysis.

Statistical analysis for the analysis of the answers from the 8 item-specific questionnaire was done with the STATA18 software. Data from the Likert scale were treated as continuous variables [10], a non-Gaussian data distribution was assumed due to small sample size, then data were reported as median and interquartile range (IQR). Due to the small sample sized the non-parametric Wilcoxon test was used to calculate the test statistic and p-value. Statistical significance was assumed for p-value < 0.05.

## Results

Results from the 3-item questionnaire were summarized in Table 1. For question n.1, two senior Otolaryngologysts (20%) answered “almost never”, meanwhile the remaining participants (80%) answered “never”. For question n.2, 5 out of 5 residents (50%) rated the VITOM^®^ Eagle “very superior” to operating microscopes in microlaryngeal surgery, meanwhile 3 out of 5 senior Otolaryngologysts (30%) rated it as “very superior” and 2 out of 5 as “superior” (20%). For question n.3, 10 out of 10 (100%) participants rated the new VITOM^®^ Eagle as “very superior” compared to VITOM^®^ 3D model for microlaryngeal surgery.

**Table 1:** Results of the 3-item questionnaire

	Seniors (*n* = 5)	Residents (*n* = 5)	Overall (*n* = 10)
Frequency of need to convert to OM*	0/0/40/60	0/0/0/100	0/0/20/80
VITOM^®^ Eagle vs. OM*	0/0/40/60	0/0/0/100	0/0/20/80
VITOM^®^ Eagle vs. VITOM^®^ 3D*	0/0/0/100	0/0/0/100	0/0/0/100

Operating microscopes (OM)

*results reported as percentage of responses to the Likert scale (1/2/3/4)

Results from the 8-item questionnaire were summarized in Table 2. Regarding the image quality and the magnification rate, the median score was 4 (IQR: 4, 4) for VITOM^®^ Eagle and 3 (IQR: 3, 3) for VITOM^®^ 3D, with a statistically significant difference (*p* = 0.002). Taking in consideration the maneuverability rate, the median score was 3 (IQR: 3, 3) for both systems, without statistically significant difference. The median score for luminance was 4 (IQR: 4, 4) for VITOM^®^ Eagle and 3 (IQR: 2, 3) for VITOM^®^ 3D, with a statistically significant difference (*p* = 0.047). The median score for both the working space and eye strain was 4 (IQR: 4, 4) for VITOM^®^ Eagle and 4 (IQR: 4, 4) for VITOM^®^ 3D, without statistically significant difference (*p* = 0.522). Instead, for stereoscopic effect, eye strain and focusing, the median score was 4 (IQR: 4, 4) for both systems, without statistically significant difference.

**Table 2:** Results of the 8-item questionnaire

Items	VITOM Eagle^1^	VITOM 3D^1^	*p*-value
Image quality	4 (4, 4)	3 (3, 3)	0.002*
Stereoscopic effect	4 (4, 4)	4 (4, 4)	-
Magnification rate	4 (4, 4)	3 (3, 3)	0.002*
Maneuverability	3 (3, 3)	3 (3, 3)	-
Luminance	4 (4, 4)	3 (2, 3)	0.047*
Eye strain	4 (4, 4)	4 (4, 4)	0.522
Working space	4 (4, 4)	4 (4, 4)	0.522
Focusing	4 (4, 4)	4 (4, 4)	-

^1^Data expressed as median and IQR in brackets.

## Discussion

This study has demonstrated the superiority of the exoscopic devices respect the traditional OM and, regarding the comparison between the first VITOM^®^ 3D and the new VITOM^®^ Eagle, the appreciation of the technological advances of the second one. Results for VITOM^®^ 3D were comparable to findings in De Virgilio et al. [2].

**Figure 1:**
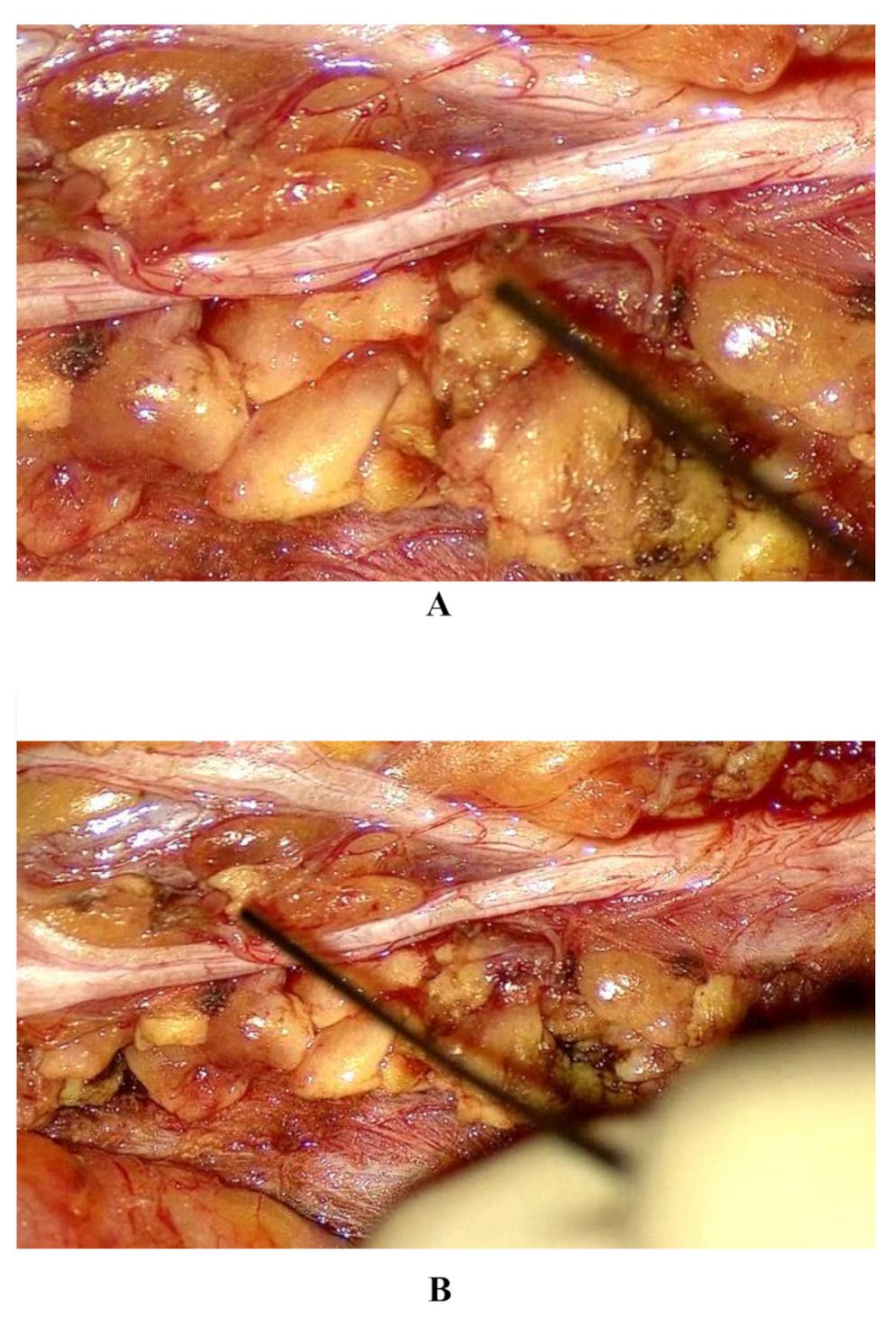
Figure 1 A Marginal mandibular branch of facial nerve at 45x magnification; B Marginal mandibular branch of facial nerve at 30x magnification

**Figure 2:**
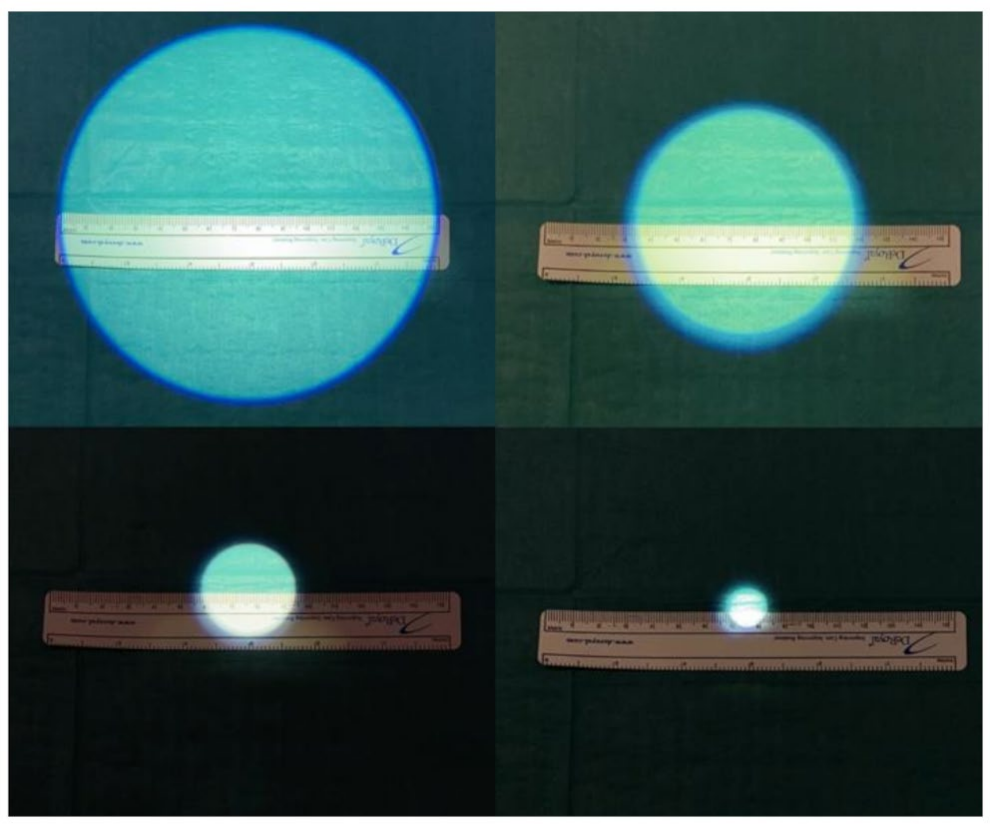
Figure 2 Adjustable light diameter

**Figure 3:**
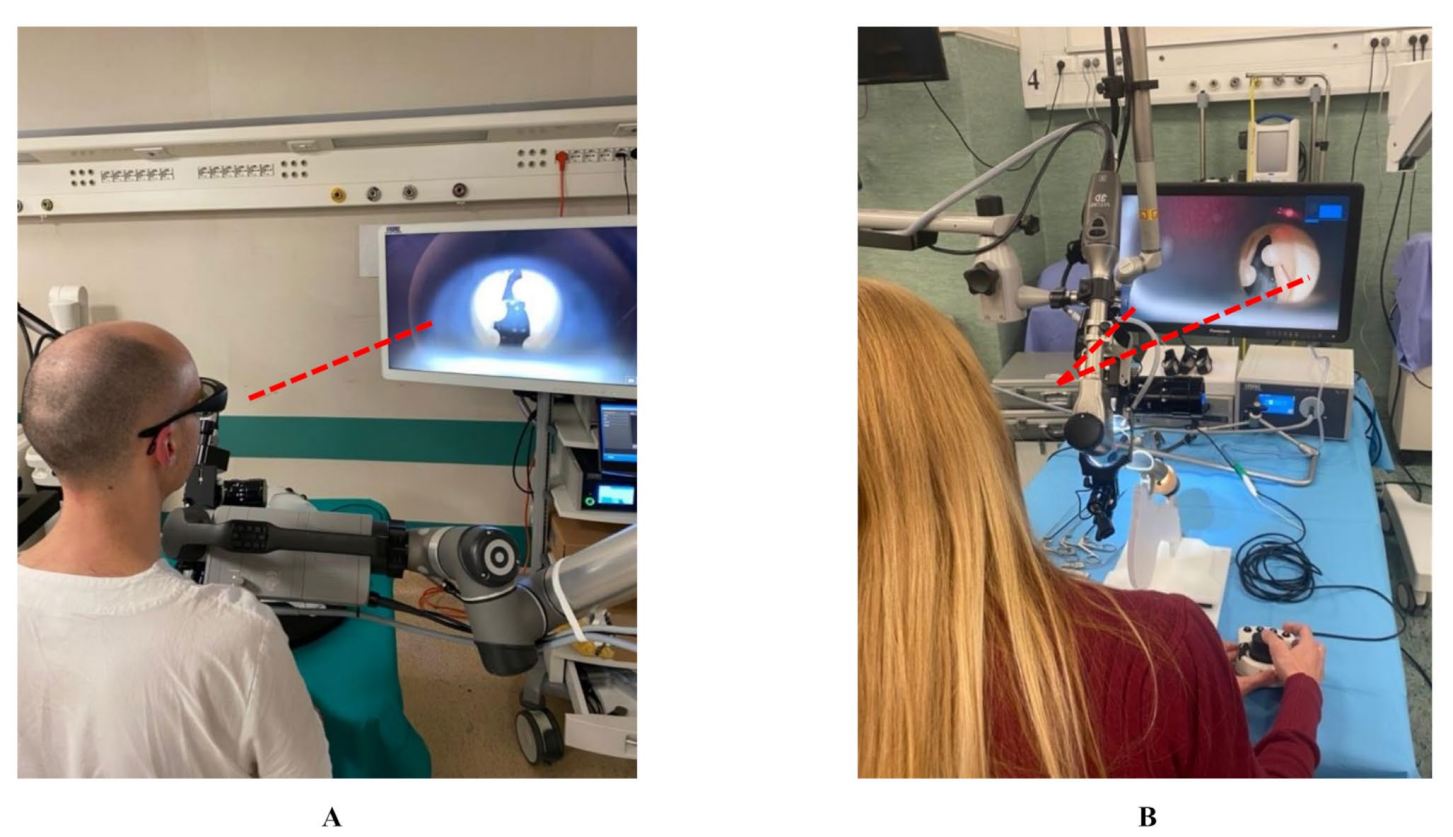
Figure 3 VITOM® Eagle allows lower positioning avoiding obstruction of surgeon’s view

The new camera features released by the Karl Storz company are multiple and, together with the ARTip™ cruise robotic system, they unlock all-new possibilities in exoscopic applications. The superiority in image quality and magnification rate is accounted by the enhanced 3D camera allows for up to 45.5x magnification (compared to the 8-30x of VITOM^®^ 3D) and offers the ability to switch between 6x optical zoom and 2x digital zoom (Fig. 1A shows the marginal mandibular branch of the facial nerve at 45x magnification during superficial parotidectomy Fig. 1B shows the marginal mandibular branch of the facial nerve at 30x magnification during superficial parotidectomy). This advanced imaging quality provides detailed visualization of even the smallest structures, offering significant advantages in different procedures including microvascular anastomosis, transoral oropharyngeal and laryngeal surgeries, middle ear surgery and otoneurosurgery. Differently from the VITOM^®^ 3D, the VITOM^®^ Eagle image can rotate around a central axis, allowing for horizontal alignment. When connected to the ARTip™ cruise robotic system, it offers translational movement along the axial plane as well as pivoting around a fixed point. These adjustments, controlled by the IMAGE1 PILOT joystick and foot pedal, optimize exposure of the oro-hypopharyngeal and laryngeal regions, enabling access to critical areas such as the lateral hypopharyngeal wall, the laryngeal commissure, and the ventricle. However, the maneuverability scores did not achieve a median score of 4; this evidence could be explained with the fact that, the ARTip™ remains a slightly bulky system to operate, even if it provides clear advantages over the Versacrane™. The statistically significant difference found in luminosity features is due to the updates not only in the type of light available but also thanks to the adjustable diameters of light beam. The latter is crucial in laryngeal surgery, in VITOM^®^ Eagle there are 7 selectable diameters (15 cm, 13 cm, 10.5 cm, 8 cm, 6 cm, 3.5 cm, 1.5 cm) (Fig. 2 shows the adjustable diameter of light), this feature is particularly useful with laryngoscopes that have narrower lumens, as it reduces light reflection on the instrument walls, improving visibility in areas such as the anterior commissure. In addition to standard white-light imaging, the VITOM^®^ Eagle offers three new modes for fluorophore-based visualization in the surgical field [11]: (1) Near-Infrared (NIR): This mode provides bright 4 K 3D visualization with indocyanine green (ICG) fluorescence, which enhances the illumination of vascular structures, assessment of tissue perfusion and tumor margins delineation, powered by a dedicated LED (Power LED Rubina^®^); (2) Blue Light Imaging (BLI): This mode helps distinguish tissues that accumulate specific fluorophores (like 5-aminolevulinic acid) with a dedicated blue light LED (Power LED Saphira^®^). This fluorescence-guided technology offers high contrast and illumination, improving precision during dissection, early detection, and localization of squamous cell carcinoma, as well as intraoperative margin assessment; (3) Fluorescein-Guided: Using the Power LED Saphira^®^ light, this technique allows for precise localization of tumor boundaries, enabling fluorescein-guided excision. VITOM^®^ Eagle also offers enhanced compatibility with a free-beam CO₂ laser micromanipulator, designed for use in transoral laser surgery, accounting for better ergonomics and faster set-up times compared to VITOM^®^ 3D. The improved ergonomics, micromanipulator interface and ARTip™ interface, allow a lower position of the system during microlaryngeal surgery preventing partial obstruction of surgeon’s monitor view as shown in Fig. 3A (shows how VITOM^®^ Eagle allows lower positioning avoiding obstruction of surgeon’s view) and Fig. 3B (shows how VITOM^®^ 3D when coupled with micromanipulator may obstruct surgeon’s view). However, results from this study demonstrate no significant differences in working space, eye strain, stereoscopic effects and focusing, as these features remained identical in the second generation of VITOM^®^ exoscopes.

In our clinical practice this updated system has already provided crucial benefits in numerous procedures ranging from oropharyngectomies to supraglottic laryngectomies, microvascular anastomoses, otological procedures and open surgeries.

The different answers reported by young surgeons versus senior ENTs highlight how the era of exoscopic technology is significantly affecting the training of future Otolaryngology specialists. Older surgeons are slightly more reluctant to a complete transition to exoscopic technology for laryngeal surgery when compared to younger surgeons, this may be due to the higher level of comfort with operating microscopes that comes from the extensive training already received on them, also taking into consideration the potential learning curve involved with new exoscopic systems. Time is the only variable capable of defining whether exoscopic technology will completely replace operating microscopes in micro-laryngeal surgery or not.

## Conclusion

Results from this study demonstrated how the new product released by Karl Storz company is considerably superior to the older VITOM^®^ 3D. The upgrades in image quality, magnification, luminance, ergonomics and interface with both the micromanipulator and the ARTip™, account for enhanced surgical visualization and precision, potentially leading to better surgical outcomes. Interestingly it was found that younger surgeons consider exoscopic technology in micro-laryngeal surgery superior in every aspect to operating microscopes. Instead, older surgeons may be slightly more hesitant to a complete transition to exoscopy, as they already carry extensive experience and comfort in the use of operating microscopes.

## Electronic supplementary material

Below is the link to the electronic supplementary material.

Supplementary Material 1
