# Supplementary material for: First experience with VITOM eagle in micro-laryngeal surgery on 3D-printed laryngeal models: important improvements in exoscopic technology
Source: Eur Arch Otorhinolaryngol. 2025 Mar 14;282(8):4211–5. doi: 10.1007/s00405-025-09293-0 (PMC12399733; doi:10.1007/s00405-025-09293-0)
Supplement: Supplementary file 1 — Supplementary Material 1 [file 405_2025_9293_MOESM1_ESM.pdf]

Questionnaires simulation day

Cross the answer you believe to be the most appropriate:

1. How many times have you felt the need to convert the procedure to an operating microscope always/frequently/almost-never/never?
2. Is the VITOM® Eagle inferior/equal/superior/very superior to operating microscopes in micro-laryngeal surgery?
3. Is the VITOM® Eagle inferior/equal/superior/very superior to VITOM® 3D in micro-laryngeal surgery?

Cross the box:

**Exoscope evaluation**	1	2	3	4
Image quality				
Stereoscopic effect				
Magnification rate				
Manouvreability				
Luminance				
Eye strain				
Working space				
Focusing				

1=Not acceptable; 2=acceptable; 3=good; 4=very good
